# Green synthesis of ZnO, MgO and SiO_2_ nanoparticles and its effect on irrigation water, soil properties, and *Origanum majorana* productivity

**DOI:** 10.1038/s41598-022-09423-2

**Published:** 2022-04-06

**Authors:** Doaa Eissa, Rehab H. Hegab, Ahmed Abou-Shady, Yousra H. Kotp

**Affiliations:** 1 Water Resources and Desert Soils Division, Soil Physics and Chemistry Department, Desert Research Center, El-Matariya, Cairo, 4540031 Egypt; 2 Laboratory of Water & Soil Chemistry, Water Resources and Desert Soils Division, Desert Research Center, El-Matariya, Cairo, 4540031 Egypt; 3 Water Resources and Desert Soils Division, Soil Fertility and Microbiology Department, Desert Research Center, El-Matariya, Cairo, 4540031 Egypt; 4 Water Treatment & Desalination Unit, Hydrogeochemistry Department, Desert Research Center, El-Matariya, Cairo, 4540031 Egypt

**Keywords:** Environmental sciences, Chemistry, Nanoscience and technology

## Abstract

The synthesis of different metal oxide nanoparticles (NPs) (e.g., ZnO, MgO and SiO_2_) using green methods is a promising alternative to traditional chemical methods. In this work, ZnO, MgO, and SiO_2_ NPs were prepared using lemon peel extract. The synthesized NPs were characterized using Fourier transform infrared spectroscopy, UV–Visible spectroscopy, X-ray diffraction, and transmission electron microscopy. Also, the effects of the green synthesis of different NPs on the irrigation water quality, the availability of some heavy metals in soil and plants, and the productivity of *Origanum majorana* (marjoram) were studied in detail. The obtained results showed that the addition of the NPs resulted in noticeable variations in the removal percentages of Cu^2+^ and Fe^3+^ from aqueous solutions. The maximum values obtained for the adsorption of Cu(II) on ZnO, MgO, and SiO_2_ NPs within the pH values of 3–5 were 89.9%, 83.3%, and 68.36%, respectively. Meanwhile, the maximum adsorption values of Fe(III) at pH 3.3 were 82%, 80%, and 65% for ZnO, MgO, and SiO_2_ NPs, respectively. Clearly, the application of the NPs effectively reduced the available Cu^2+^ in the studied soil samples in the following order: Zn2 > Zn1 > Mg2 > Si2 > Mg1 > Si1 > C (control). The highest values of available Cu^2+^ were observed in the control treatment, whereas the lowest values were obtained when Zn2 was added. The same tendencies were observed with substantial concentrations of Fe. The addition of NPs to the soil samples positively affected the plants' Cu^2+^ uptake. The effects of NPs and the additions of Cu^2+^ and Fe^3+^ on the availability of nitrogen, phosphorus, and potassium (NPK) in the soil system were very completed and osculated from one treatment to another. The same tendencies were observed with the total concentration of NPK in plants.

## Introduction

Recently, different studies in various fields of interest have been focused on enhancing the use of different materials on very small scales close to those of molecules and atoms. Generally, 1 nm is calculated as one billionth of a meter and is considered approximately ten times the diameter of a hydrogen atom. It is known that at nanometer sizes, the chemistry and physics of materials are not expected to be appropriate. These include the reactivity, strength, color, and conductivity of materials, which substantially differ between nanoscales and largescales^1,2^. In the past years, the field of green chemistry has been a great area of interest, particularly in the context of a continuous energy crisis. Nanoscience may be the appropriate field in this dimension as it enables many biochemical, chemical, and biophysical transformations through reliable and easy means^3^. Thus, the green synthesis of ZnO, MgO, and SiO_2_ NPs has been exploited in several fields such as cancer treatment^4^, environmental remediation^5^, environmental and biomedical field applications^6,7^, transportation, food safety, environmental science, sustainable energy, medicine, and catalysis^8^.

In farming, nanotechnology has been exploited as a potential solution for reshaping farming construction. This could be done by replacing traditional materials used for farming construction such as pesticides, herbicides, and fertilizers with their nanoscale counterparts. Such nanoscale materials have been applied to several agricultural practices (e.g., soil foliar applications) and for grain and leafy plants^8,9^. Also, researchers adopt green technologies in preparing different nanoparticles (NPs) for medicinal uses in response to the increased demand for environmentally friendly NPs^10^. Chemical synthesis processes such as chemical vapor deposition, micellization, sol–gel formation, chemical precipitation, pyrolysis, hydrothermal methods, and others frequently result in poisonous chemicals adsorbed on surfaces, which might have negative consequences in medical applications. Some reactions necessitate high temperatures and/or high pressures to start, whereas others require inert atmospheres and/or the use of toxic substances such as H_2_S, toxic templates and stabilizers, and metallic precursors^11^. Moreover, the chemicals utilized in the formation and stabilization of NPs are hazardous and produce non-ecofriendly byproducts^12^. Thus, biological processes involving microbes, plants, and plant extracts have been proposed as viable alternatives to chemical procedures for preparing ZnO, MgO, and SiO_2_ NPs.

ZnO, MgO, and SiO_2_ NPs have been synthesized using various biological systems comprising bacteria, fungi, and yeasts^6,8,13^. Microorganism-supported NP manufacturing entails complex processes involving cell culture maintenance, intracellular synthesis, and numerous purification processes. In this regard, because standard chemical procedures are costly and necessitate the use of chemical mixes/organic solutions that act as plummeting agents, adopting “green” approaches in manufacturing zinc oxide NPs has developed into a growing area of interest^14^.

Several materials utilized as fertilizers are considered nanomaterials, including nano-CaO, nano-urea, nano-hydroxyapatite (nano-HAP), Mg NPs, and MgO NPs. These are considered the most widespread macronutrients reported for improving biomass construction and plant expansion. For instance, studies that used nano-HAP and nano-Mg reported increases in seed yield production for soybean (*Glycine max*) and cowpea (*Vigna unguiculata*). The study of Liu and Lal^15^ reported augmented growth (32.6%) and yield (20.4%) of soybean vegetation when soils were modified using nano-HAP (21.8 mg l^−1^). In contrast, when the vegetation was exposed to the same concentration of Ca(H_2_PO_4_)_2_ as the main source of phosphorous, the obtained values were moderately small. The study presented by Aziz et al*.*^16^ showed that synthesized composites with different concentrations of nitrogen, phosphorus, and potassium (NPK) (e.g., 50, 60, and 400 mg kg^−1^) minimized the life cycle of nanofertilized wheat plants compared with those fertilized with regular nutrients contained in the form of polymers (chitosan). Wheat (*Triticum aestivum*) plants fertilized with chitosan NPs containing NPK via foliar application had a shortened harvesting duration (130 days) after planting compared with 170 days after planting if normal fertilizers containing NPK were used.

Several scientists described the benefits of engineered nanomaterial formulations with pesticide properties^17,18^. The study conducted by Chhipa^18^ showed that the pesticide properties of silver NPs within major components presented a noticeable influence against numerous fungal species. In contrast, when Cu NPs were utilized, the efficiencies of the applications against bacteria and fungi were remarkably improved. Other several materials such as hezaconazole and nanosulfur also presented high efficiencies as fungicides. The only constraints when using such nanomaterials as pesticides are their ecological impacts, which are not well understood yet^18^. Adisa et al.^17^ studied polymer-support nanopesticides such as nanogels, nanospheres, nanofibers, and nanocapsules classified as antimicrobials materials that possess different environmental persistence abilities. Generally, nanospheres can store active components dispersed throughout nanomatrices; however, their active ingredients are encased in the polymeric matrix^17^.

On another note, the water problem has recently become a global issue, and water reuse could be one of the means of reducing the strain on already depleting water resources^19^. The reported causes of domesticated water reprocessing were established to be osculated from one region to another (e.g., 32% in Asia and 51% in northern Europe for environmental purposes; 46% in California for agriculture; 7% in Japan for agriculture; 44% in Florida, 25% in Tunisia, and 4% in Australia for total consumption; 25% in Spain for agriculture; and 75% in Israel for agriculture. Moreover, generally 500 Mm^3^ year^−1^ wastewater used in Mexico and China were directly reused without treatment^20–22^. Consequently, inorganic contaminants may accumulate in irrigation water or soils. Thus, the novelty in this work is to synthesize ZnO, MgO and SiO_2_ NPs using a low-cost and safe method. Also, it studied the effects of these NPs on the quality of irrigation water, the contents of some heavy metals in soils and plants, and the productivity of *Origanum majorana* (marjoram).

## Material and methods

### Materials

Lemon (*Citrus* *limon*)^23^ peel extracts were used for the green synthesis of MgO, SiO_2_, and ZnO NPs. The magnesium nitrate, zinc sulfate, and sodium metasilicate possessed high purity (≥ 98%). All materials were purchased from Merck Chemicals Ltd., whereas the fresh peels of lemons were collected from different lemon farms in Egypt. The fresh peels were first washed with distilled water and then soaked in an ethanol (C_2_H_5_OH) and ammonium hydroxide (NH_4_OH) solution purchased from Merck Chemicals Ltd., Darmstadt, Germany.

### Preparation of MgO, SiO_2_, and ZnO NPs

MgO, SiO_2_, and ZnO NPs were prepared in two main steps: (1) the collection of lemon peel extract and (2) the synthesis of MgO, SiO_2_, and ZnO NPs.

#### Preparation of lemon peel extract

The lemon peels were washed with distilled water several times to remove any dust and then dried at 60 °C inside a furnace for 48 h. The dried lemon peels were ground, crushed, and sieved into a suitable size. The extract was then obtained at a concentration of 250 g L^−1^ at 25 °C for 24 h with steady shaking. Thereafter, the extract was filtered using a filter paper.

#### Synthesis of MgO, SiO_2_, and ZnO NPs

For preparing MgO, SiO_2_, and ZnO NPs, we added 0.5 mol L^−1^ of magnesium nitrate, sodium metasilicate, and zinc sulfate aqueous solution to the lemon peel that was separated previously from the extraction solution. The solution was then boiled to 70 °C for 1 h until it was reduced into a white-colored solution. This color change is considered a mark of the formation of soluble fractions from the peel extract. It was demonstrated that the ethanol lemon peel extract contained hesperidin flavanol, which discharges aglycone that may be used as a reducing agent^24,25^. The pH of each NP mixture was adjusted through the addition of ammonium hydroxide solution (0.1 mol L^−1^ to maintain the pH values of 9.7, 11.58, and 6.95 for MgO, SiO_2_, and ZnO NPs, respectively. Afterward, the precipitates were washed several times with deionized water, centrifuged and collected in a clayey crucible, and heated in a muffle at 500 °C for 3 h for both MgO and ZnO NPs and at 700 °C for SiO_2_ NPs. Elevating the temperature in the muffle was mandatory to remove any organic residuals. Finally, a white precipitate for each NP was obtained and packed carefully for further characterization. The probable mechanisms for the production of MgO, ZnO, and SiO_2_ NPs are defined below:

1 $${\text{Mg}}\left( {{\text{NO}}_{{3}} } \right)_{{2}} + {\text{lemon peel extract }} \to {\text{ Mg}}\left( {{\text{OH}}} \right)_{{2}}$$

2 $${\text{Mg}}\left( {{\text{OH}}} \right)_{{2}} + {\text{calcination}} \to {\text{ MgO}} + {\text{H}}_{{2}} {\text{O}}$$

3 $${\text{ZnSO}}_{{4}} + {\text{lemon peel extract }} \to {\text{ Zn}}\left( {{\text{OH}}} \right)_{{2}}$$

4 $${\text{Zn }}\left( {{\text{OH}}} \right)_{{2}} + {\text{calcination}} \to {\text{ ZnO}} + {\text{H}}_{{2}} {\text{O}}$$

5 $${\text{Na}}_{{2}} {\text{SiO}}_{{3}} + {\text{lemon peel extract }} \to {\text{ Si}}\left( {{\text{OH}}} \right)_{{4}}$$

6 $${\text{Si}}\left( {{\text{OH}}} \right)_{{4}} + {\text{calcination}} \to {\text{ SiO}}_{{2}} + {\text{2H}}_{{2}} {\text{O}}$$

### Characterization of the prepared nanoparticles

The MgO, SiO_2_, and ZnO NPs were characterized using the following procedures. Powdered NPs were analyzed using X-ray diffraction (XRD) (PAN Analytical X'Pert TopScore Plus Diffractometer) operated at 40 kV with a current of 30 mA under Cu-Kα radiation of a 2*θ* range of 10°–80°. The surface morphologies of the NPs were observed using transmission electron microscopy (TEM) with an elevated-resolution (JEOL JEM-1400 UHR, operated at 80 kV). The chemical structures of the prepared NPs were investigated using Fourier transform infrared spectroscopy (FTIR) infrared spectra (using a Nicolet Avatar 230 spectrometer. UV–Vis spectrophotometer between 200 and 800 nm wavelength range (Elico EI 301E, India) was used for scanning different prepared nanoparticles.

### Soil sampling and analysis

Soil samples were collected from a constant depth of 0–30 cm from the El-Gabal El-Asfar area, Egypt. Some soil and water properties are listed in Table 1. The soil samples were air-dried, crushed, and sieved through a 2-mm sieve to ensure the removal of gravel and organic waste. The soil texture was determined following the international pipette method. The other properties of the soil samples, including organic matter content, pH, and electrical conductivity, were determined according to published literature^25–27^. The heavy metal contents were determined using inductively coupled argon plasma optical emission spectrometry (ICAP 6500 Duo, Thermo Scientific, England). N, P, and K were determined in an acid digested solution, which was prepared according to Dahnke and Johnson^28^. The available nitrogen in the soil samples was extracted using a 2M potassium chloride solution and determined according to^29^. The available potassium and phosphorous were extracted using a DTPA^+^ ammonium bicarbonate solution and measured following the method described by Soltanpour^30^.

**Table 1:** Some physical and chemical properties of El-Gabal El-Asfer soil samples and irrigation water.

Soil properties	El-Gabal El-Asfer soil	Water properties	Tap water	El-Gabal El-Asfer irrigation water
Particle size distribution (%)	80.30	pH	7.30	8.42
Sand (%)	7.90			
Silt (%)	11.80	EC (dS m^−1^)	0.84	1.57
Clay (%)				
Texture class	Loamy sand	Cation and anion (mg dm^–3^)		
		Ca^++^	15.30	3.93
		Mg^++^	80.10	3.25
		Na^+^	41.20	16.81
		K^+^	4.00	0.36
CaCO_3_ (%)	9.78	HCO_3_^−^	34.74	8.03
OM (%)	2.81	CO_3_^–^	0.00	1.31
CEC (meq 100 g^−1^)	20.70	Cl^−^	48.95	11.41
pH (1:2.5)	8.01	SO_4_^–^	21.13	3.60
EC (dS m^−1^)	2.78			
Total content of heavy metal (mg kg^−1^)		Heavy metal (mg kg^−1^)		
Fe	28,742	Fe	–	0.04
Cu	6.92	Cu	–	0.03
Chemically extractable heavy metals (mg kg^−1^)				
Fe	13.24	–	–	–
Cu	4.55	–	–	–

### Adsorption experiments

Series of experiments was conducted to investigate the effects of different pH levels on the removal of Cu^2+^and Fe^3+^ ions using MgO, SiO_2_, and ZnO NPs as adsorbent materials with an initial concentration of 50 mg L^−1^ for both Cu^2+^ and Fe^3+^. The solid to liquid ratio was 10 g L^−1^ the shaking rate was 175 rpm, and the temperature was adjusted to 25 °C. Solutions with different pH values were prepared, and the pH was adjusted by adding nitric acid (0.1 M mol). After the prevailing 24 h of adsorption, the concentrations of the remaining Cu^2+^ and Fe^3+^ ions in each solution were detected using inductively coupled argon plasma optical emission spectrometry (ICAP 6500 Duo, Thermo Scientific, England).

The adsorption isotherm of Cu^2+^ and Fe^3+^ was obtained using the prepared NPs as adsorbents, in which a series of concentrations of Cu^2+^ and Fe^3+^ was prepared by dissolving copper sulfate and iron chloride with an initial concentration of copper and iron ranging from 20 to 500 mg L^−1^. The initial pH values for the Cu^2+^ and Fe^3+^ solutions were 5.2 and 3.2, respectively. Generally, 50 ml of each solution and 0.5 g of the adsorbent were mixed in a 100-ml sealed conical flask shaken at a constant speed of 175 rpm in a thermostatic water bath at 25 °C. After 24 h of adsorption, the concentrations of the remaining Cu^2+^ and Fe^3+^ in each solution were determined.

### Pot experiments

Pot experiment was carried out in green house of Desert Research Center, Egypt. located at 30° 7.29′ N, 31° 18.93′ E. Pot experiments were conducted during the cropping season of 2020–2021 to investigate the effects of MgO, SiO_2_, and ZnO NPs on plant yield and the availability of some heavy metals in both the soil and marjoram hat were polluted at three levels with either Fe^3+^ or Cu^2+^. Marjoram was planted in pots containing 18 kg of soil after approximately 30 days from seed germination. Fertilizer doses were added to all treatments (as ammonium sulfate, calcium superphosphate, potassium sulfate, and biofertilizers)^31^. The experimental design was factorial with three replicates at three levels (0, 5, and 10 mg kg^−1^) of each pollutant (Cu^2+^ and Fe^3+^) and at 0, 150, and 300 mg kg^−1^ of each of the MgO, SiO_2_, and ZnO NPs. A sum of 35 treatments was performed after 30 and 60 days from the transplanting date (April 21 and May 23). The plants were irrigated twice a month with 55 cm^3^ for each treatment. After 97 days from the planting, the marjoram plants were cut at the soil surface and washed with deionized water. The plants were oven-dried at 70 °C for 48 h, weighed for dry matter yield, and ground. The plants and soil samples of the different treatments were digested using H_2_SO_4_–H_2_O_2_^32^. The heavy metal contents were detected using ICAP.

### Statistical analysis

The data obtained in the present work were statistically analyzed, and the differences between the means of the different treatments were considered significant when they were greater than the values of the least significant difference at the 5% level using the Statistic program version No. 9. Alltreatments were used in a factorial design.

### Statement

The collection of lemon peel and the cultivated *Origanum majorana* samples was done by getting the permission from the local suppliers. Te authors confirm that all methods were performed in accordance with the relevant guidelines and regulations.

## Results and discussion

### Characterizations of the NPs

**Figure 1:**
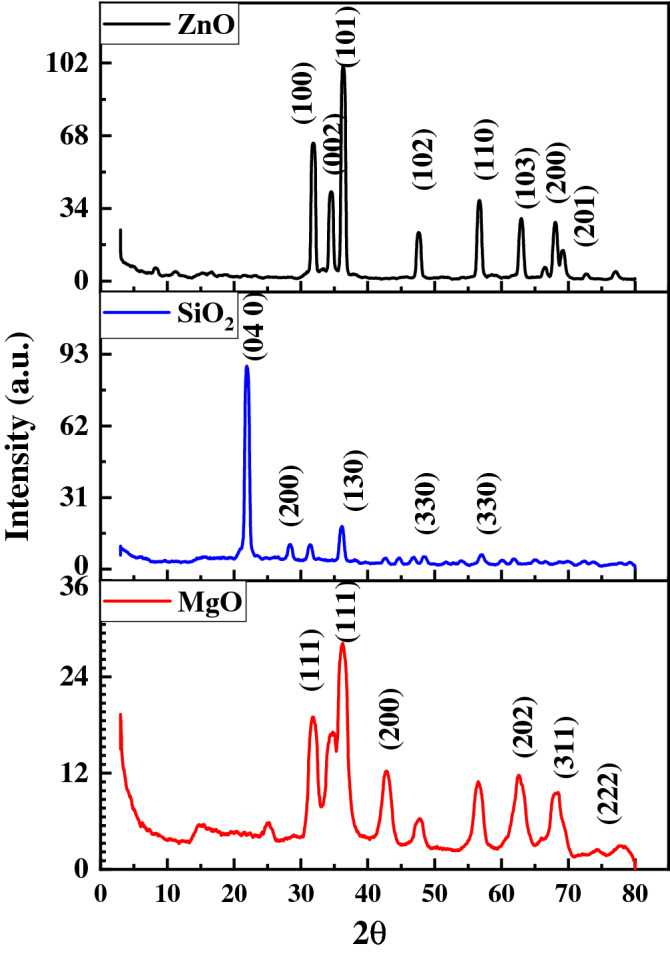
The X-ray diffraction profile of NPs synthesized using the lemon peel extract.

XRD is a widely used method for determining particle size and NP structure. The XRD of the different NPs produced with the lemon peel extract is shown in Fig. 1. The following is how the Debye–Scherrer equation was used to determine the dimensions of the MgO, SiO_2_, and ZnO NPs:

7 $$D = K\lambda /(\beta {\text{cos}}\theta )$$

where *D* is the crystal volume, *λ* is the wavelength of the X-ray radiation (*λ* = 0.15406 nm) for Cu-Kα,*K* is frequently taken as 0.9, and *β* is the line width at half-maximum height. The peaks of the MgO NPs matched with the plane (JCPDS card No. 89–7102) and were found at 2*θ* = 36.72° (111), 2*θ* = 42.80° (200), 2*θ* = 62.38° (202), 2*θ* = 74.5° (311), 2*θ* = 78.5° (312), (222). The MgO NPs produced with lemon peel extract had a crystallized face-centered cubic (fcc) phase of magnesium oxide, according to the XRD spectrum^33^. Using Scherrer's formula, the crystal size was predicted to be around 16.77 nm. The SiO_2_ NPs had peaks that correspond to the planes 2*θ* = 28.4° (040), 2*θ* = 31.5° (200), 2*θ* = 36.06° (130), 2*θ* = 48.4° (330), and 2*θ* = 56.9° (330)^34^. The XRD spectra indicate that the SiO_2_ NPs made from the lemon peels were crystalline. Using Scherrer's formula, the calculated crystal diameters were around 42.6 nm. In addition, the ZnO NP patterns also suggest that the planes were approximately 2*θ* = 31.8° (100), 2*θ* = 34.4° (100), (002), 2*θ* = 36.3° (101), 2*θ* = 47.5° (102), 2*θ* = 56.6° (110), 2*θ* = 56.69° (110), 2*θ* = 62.8° (103), 2*θ* = 67.98° (200), and 2*θ* = 69.1° (200), (201). These findings are in line with those of other researchers who have shown ZnO diffractograms^16,35^. As indicated in Fig. 1, no contaminant diffraction peaks were identified. These findings revealed that the produced ZnO was extremely pure. No usual graphitic carbon diffraction peaks were found. Because graphitic carbon is naturally amorphous, its existence cannot be ruled out. The sample's highly crystalline nature is indicated by the crisp and high diffraction peaks^36^. High heat treatment is likely to provide sufficient kinetic energy for rearranging atomic groupings and hence the development of NP crystal structures^37^. ZnO was synthesized in this process at a sufficiently high temperature of around 500 °C, resulting in a product with a crystalline structure. Using Scherrer's formula, the crystallite size of the ZnO nanocrystallite was around 60.5 nm.

**Figure 2:**
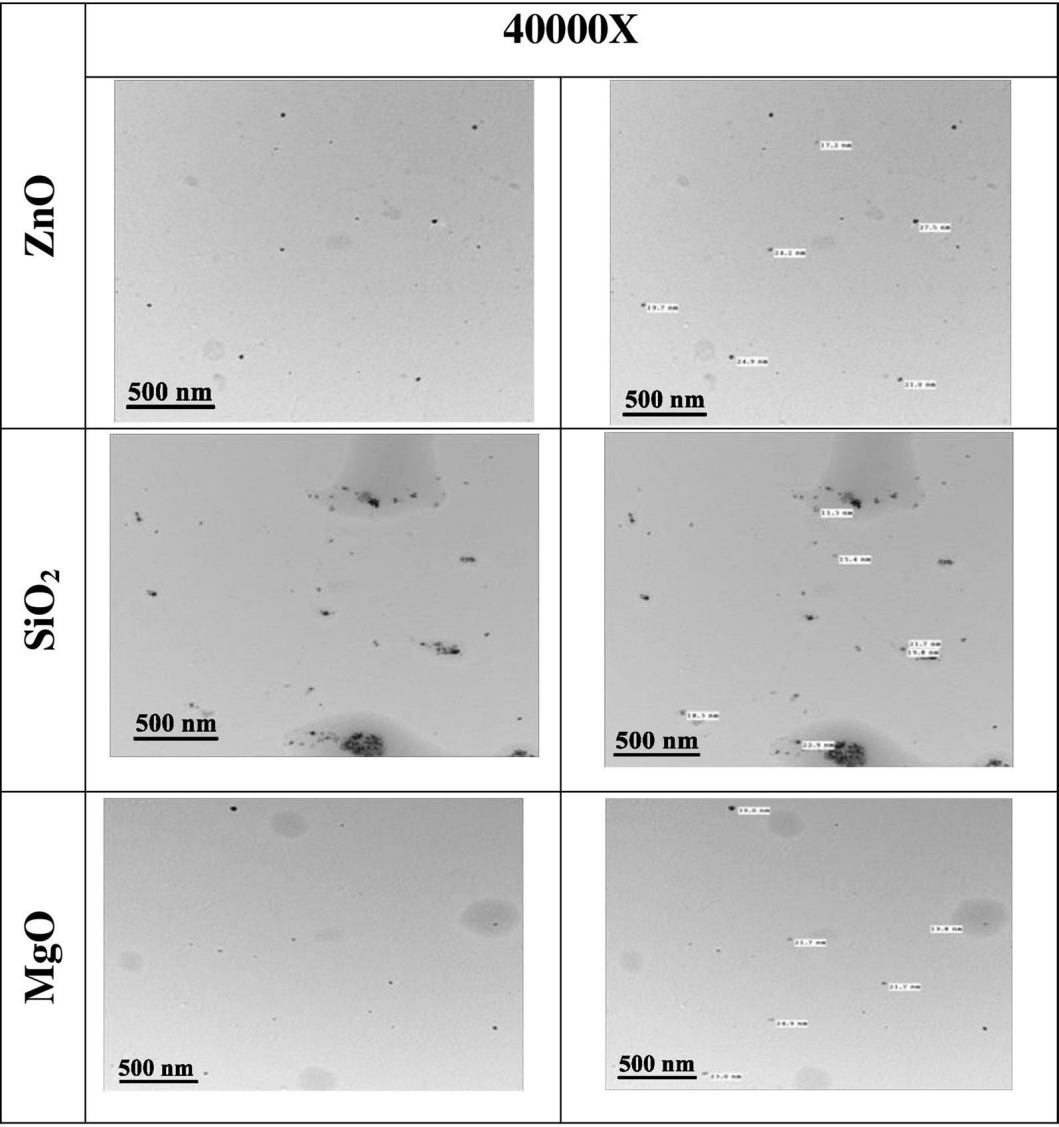
The TEM micrograph of MgO, SiO_2_, and ZnO nanoparticles synthesized by lemon peel extract.

**Figure 3:**
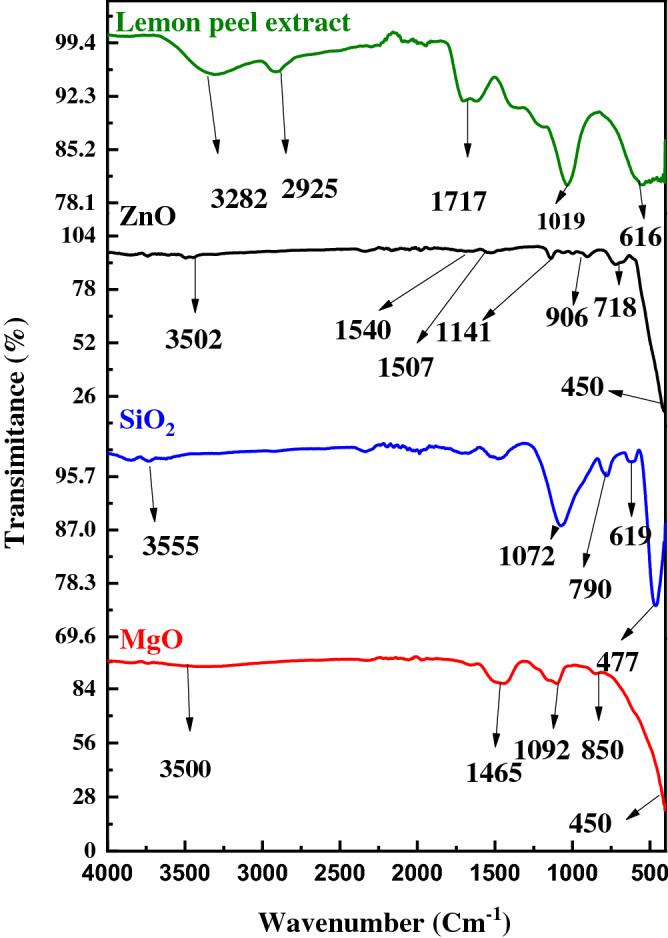
The FTIR spectra of the MgO, SiO_2_, and ZnO nanoparticles and lemon peel extract.

TEM images provide a more detailed insight into the interior structure of NPs. Figure 2 shows a TEM image of the MgO, SiO_2_, and ZnO NPs produced with lemon peel extract. The circular structures are built of unique individuals of the distinct manufactured NPs, according to the increased two-dimensional picture of the NPs. Each produced NP has a size of about less than 100 nm, as measured by the TEM imaging. This size corresponds to that calculated from the XRD pattern. The FTIR technique was used to determine the probable chemical bonds in the lemon peel extract and the produced NPs. The FTIR spectra of the MgO, SiO_2_, and ZnO NPs, as well as that of the lemon peel extract, are shown in Fig. 3. The spectrum of the lemon peel extract had a strong peak at around 3200–3700 cm^−1^, which may be attributed to the stretching mode of the hydroxyl of the phenols, which also coincide with the N–H of the amines, which could be attributable to the various bioactive chemicals found in lemon^38,39^. The symmetrical and asymmetrical C–H of the aliphatic groups has been assigned to two minor intensity peaks at about 2925 cm^−1^. The bending vibration of the OH group was attributed to a wide broad peak at about 1717 cm^−1^, which could be due to chemisorbed and/or physisorbed moisture on the surface of the NPs^40^. The occurrence of C–O symmetric stretching of alcohol and vibrating C–H of the –CH=CH of the ethylene structure can be seen in the bands at 1019 and 616 cm^−1^, respectively. When these peaks are compared with the spectra of the MgO NPs, a decrease in the peak position of the MgO NPs is observed. The MgO NPs had an absorption band at 450–850 cm^−1^ in the FTIR spectrum. The O–H stretch manifested as a very broad band in the spectra, spanning 3700 cm^−1^^41,42^. The saturated main peak between 1092 and 1456 cm^−1^ indicate significant concentrations of carbon and are also characteristic of the ligand^40^. Glycone steroids may have played a key role in the stabilization and capping of MgO NPs in our research^25,38,39^. When these peaks are compared with the spectra of the ZnO NPs, the peak broadening of the ZnO NPs is reduced. The typical Zn–O stretching vibration of the ZnO NPs caused a significant peak at about 450 cm^−1^^43,44^. Before the calcination of the intermediate product to ZnO NPs, biomolecules were responsible for the conversion of ZnSO_4_ to Zn(OH)_2_.

**Figure 4:**
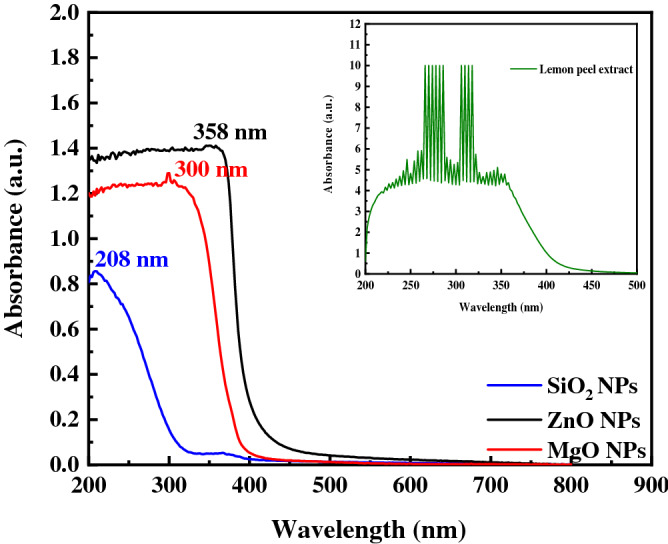
UV–Vis spectrophotometer of lemon peel extract and different prepared NPs.

Figure 3 shows the FTIR spectra of the produced SiO_2_NPs. Si–O has a band at around 790 cm^−1^, and the symmetric stretching and bending vibrations of Si–O–Si have bands at around 477 and 619 cm^−1^, respectively^44^. Bending vibration occurs when oxygen moves at a perfect angle to the Si–Si bands in the Si–O–Si plane^45,46^. The stretching vibration of the Si–O–Si band, wherein the bridging oxygen atom shifts parallel to the Si–Si lines in the reverse direction of their Si neighbors, corresponds to the band at approximately 1072 cm^−1^. The 3555-cm^−1^ band is specific to Si–OH hydrogen-bonded stretching modes^46^. The UV–visible spectra of lemon peel extract, as well as MgO, SiO_2_, and ZnO NPs are shown in Fig. 4. The qualitative UV‐VIS profile of ethanolic extract of *lemon peel* was taken at the wavelength of 300 nm to 500 nm owing to the sharpness of the peaks and proper baseline. The profile showed the peaks at 242, 246, 254, 258, 262, 266, 270, 274, 278, 282, 286, 290, 294, 298, 302, 306, 310, 314, 318, 322, 326, 330, 334, 338, 342, 346, 352 and 354 nm. In the UV–VIS spectra the presence of more peaks in the wavelength region from 200 to 500 nm is obvious and indicates the existence of unsaturated groups and heteroatoms compounds containing S, N, O^47^. The peaks at 354 nm indicate the presence of organic chromophores within the lemon peel extract. The peak located between 320 and 360 nm should be ascribable to the existence of phenolic mixtures^1^. Similarly, the band between 280 and 315 nm signposts to the presence of flavonoids and aloin^1^. Nevertheless, Jain et al.^47^, stated that**,** the use of UV–visible spectrophotometery in the studding of complex extract is limited by the essential difficulties in assigning the absorption peaks to any certain components in the structure. Hence, UV–VIS outcomes must be accompanied with GC/MS, to assist appropriate extract characterization and component identification^47^. The UV–visible spectra of MgO NPs are shown in Fig. 4. The absorption spectra of the reaction media with absorbance at 300 nm established the existence of MgO NPs. This verifies the reduction of Mg (NO_3_)_2_ and the emergence of MgO^48^. The absorption spectra of the green synthesized ZnO NPs exhibited maximum optical absorption band at 358 nm^1^. The green synthesized SiO_2_ nanoparticles exhibited one absorption peak at 208 nm, attributable to the characteristic absorption of SiO_2_^49^_._

### Batch adsorption experiments

#### Effects of the initial pH on the adsorption efficiency

**Figure 5:**
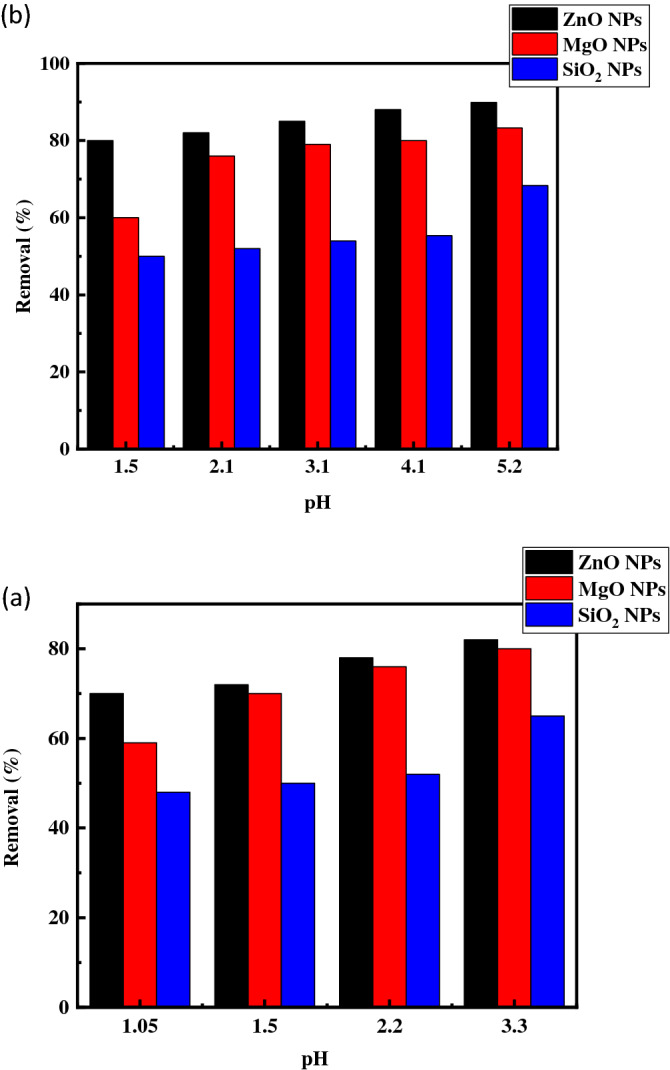
Effect of pH on adsorption of Cu^2+^ (**a**) and Fe^3+^ (**b**) ions (conditions: 10 gl^−1^ adsorbent, 10 ml of 50 mg L^−1^ of ions, duration of oscillation time of 24 h at temperature 25 °C).

The pH of any solution is considered one of its most important parameters that basically influence the adsorption of ions such as Cu^2+^ and Fe^3+^, as studied in this work. To evaluate the effects of pH on the removal efficiency of Cu^2+^ and Fe^3+^, experiments were performed in pH ranging from1 to 5 and 1 to 3.5, respectively. The initial concentrations of either Cu^2+^ or Fe^3+^ were close to 100 mg L^−1^, as shown in Fig. 5. The pH values exceeding 5 and less than 3.5 for Cu^2+^ and Fe^3+^ ions, respectively, were not studied to avoid the natural phenomenon of Cu^2+^ and Fe^3+^ precipitation in hydroxide forms. Apparently, the variations in the removal percentages with different pH values were approximately constant, and maximum values were obtained within pH values ranging from 3 to 5 for Cu^2+^, where removal percentages of 89.9%, 83.3%, and 68.36% were achieved for ZnO, MgO, and SiO_2_ NPs, respectively. Comparatively, the removal percentages were 82%, 80%, and 65% for the adsorption of Fe on the ZnO, MgO, and SiO_2_ NPs, respectively, at pH 3.3.

In general, adsorption percentages are reduced with lower pH levels. This is may be because of the reduced number of negative sites and the competition between the positive copper, iron, and hydrogen ions on these negative adsorption sites^50^. By increasing the pH of media, more adsorptive sites are created, and the adsorption capacity is increased. However, with continued increases in pH values, the formation of precipitates of Cu^2+^ and Fe^3+^ hydroxide is increased. Accordingly, adsorption experiments at pH values of 5.2 and 3.3 were preferable for Cu^2+^ and Fe^3+^, respectively.

#### Adsorption isotherm models

Adsorption isotherms were used to easily describe the adsorption of different fractions of sorbet molecules that are partitioned between liquid and solid phases at equilibrium. Generally, the adsorption of Cu^2+^ and Fe^3+^ ions on the ZnO, MgO, and SiO_2_ NPs was modeled using two adsorption isotherms.

##### Freundlich isotherm

The Freundlich isotherm is a model used to represent monolayer (chemisorption) and multilayer (physisorption) adsorption. It is founded on the supposition that molecules adsorb onto the uneven surface of an adsorbent^51^. Freundlich's equation is written in linear form as follows:

8 $${\text{log}}q_{{\text{e}}} = {\text{log}}K_{{\text{F}}} + {1}/n {\text{log}}C_{{\text{e}}}$$

**Figure 6:**
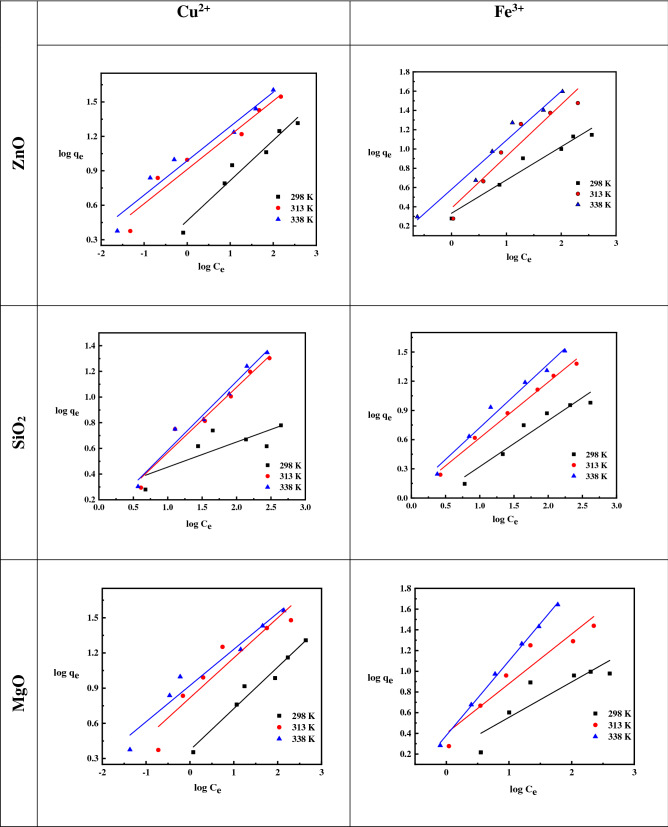
Linearized Freundlich isotherms for Cu^2+^ and Fe^3+^ ions adsorption on ZnO, MgO, and SiO_2_ NPs at different temperatures.

The Freundlich isotherm coefficients *K*_F_ and *n* are calculated first from the intercept and slope of a chart of log*q*_e_ vs. log*C*_e_, as shown in Fig. 6. In this investigation, *n* values greater than unity were discovered, indicating chemisorptions (Table 2)^52^. L-type isotherms with *n* > 1 suggest a high affinity between the adsorbate and the adsorbent and are suggestive of chemisorption^53^. With increasing temperature, the Freundlich constant (*K*_F_) shows that the adsorption mechanism is endothermic.

**Table 2:** Freundlich isotherm model parameters and correlation coefficients for the adsorption of Cu^2+^ and Fe^3+^ ions on ZnO, SiO_2_, and MgO NPs.

Freundlich isotherm model parameters										
Metal ions	Temperature	ZnO			SiO_2_			MgO		
		n	K_F_	R^2^	n	K_F_	R^2^	n	K_F_	R^2^
Cu^2+^	298	2.85	2.88	0.95	5.26	1.05	0.63	2.85	2.34	0.96
	313	3.35	8.12	0.94	2.00	1.13	0.96	2.94	6.45	0.88
	338	3.35	9.54	0.94	1.88	1.11	0.97	3.33	8.31	0.95
Fe^3+^	298	2.94	2.15	0.95	2.11	0.70	0.92	2.92	1.62	0.78
	313	1.88	2.42	0.96	1.74	1.10	0.99	2.08	2.51	0.90
	338	1.97	3.81	0.92	1.53	1.17	0.98	2.38	2.70	0.99

##### Langmuir isotherm

**Figure 7:**
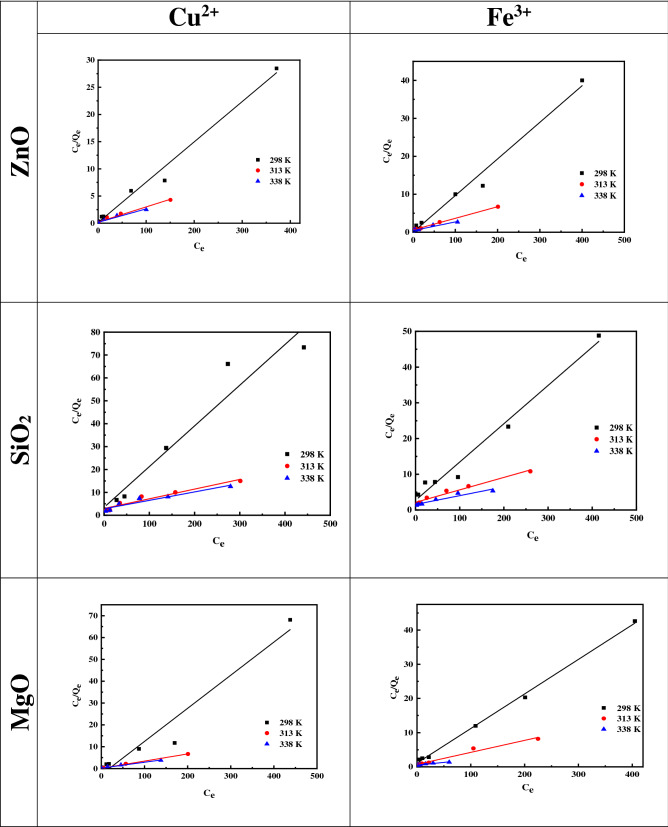
Linearized Langmuir isotherms for Cu and Fe ions adsorption on ZnO, MgO, and SiO_2_ NPs at different temperatures.

The Langmuir isotherm assumes the adsorption of a monolayer on a uniform surface with a finite number of adsorption sites. Once a site is filled, no further sorption can take place at that site as the surface will eventually reach a saturation point where the maximum adsorption of the surface will be achieved. The linear form of the Langmuir isotherm model is described as follows:

9 $$\frac{{C_{e} }}{{q_{e} }} = \frac{{C_{e} }}{{q_{\max } }} + \frac{1}{{q_{\max } K_{L} }}$$

where *K*_L_ is the Langmuir constant related to the energy of adsorption, and *q*_max_ is the maximum adsorption capacity (mg g^−1^)^54^. The slope and intercept of plots of *C*_e_/*q*_e_ versus *C*_e_ at different temperatures were used to calculate *q*_max_ and *K*_L_, as is shown in Fig. 7. The Langmuir isotherm parameter was appropriate for Cu^2+^ and Fe^3+^ adsorption on the ZnO, MgO, and SiO_2_ NPs and yielded isotherms that were in good agreement with the observed behaviors, as presented in Table 3. The Cu^2+^and Fe^3+^ adsorption capacities on the ZnO, MgO, and SiO_2_ NPs at room temperature (297 K) were respectively 135, 66, and 58 mg g^−1^ for Cu^2+^ and 104, 100, and 94 mg g^−1^ for Fe^3+^, as shown in Table 3. These are much higher than the adsorption capacities of other adsorbents reported in other relevant literature:that of activated carbon reached 3.37 mg g^−1^^55^, whereas the adsorption of hematite reached 4.94 mg g^−1^^56^.

**Table 3:** Langmuir isotherm model parameters and correlation coefficients for the adsorption of Cu^2+^ and Fe^3+^ on ZnO, SiO_2_, and MgO NPs.

Langmuir isotherm model parameters										
Metal ions	Temperature	ZnO			SiO_2_			MgO		
		q_max_ (mg g^−1^)	K_L_ (L mg^−1^)	R^2^	q_max_ (mg g^−1^)	K_L_ (L mg^−1^)	R^2^	q_max_ (mg g^−1^)	K_L_ (L mg^−1^)	R^2^
Cu^2+^	298	135.13	9.54	0.98	58.82	2.00	0.94	66.22	0.01	0.94
	313	370.37	1.61	0.97	150.57	9.12	0.94	305.81	1.38	0.99
	338	403.22	1.42	0.95	333.33	6.30	0.92	370.37	1.51	0.97
Fe^3+^	298	104.16	4.50	0.98	93.45	5.10	0.98	100.00	12.02	0.99
	313	333.33	3.98	0.99	294.11	1.41	0.97	285.71	5.12	0.97
	338	454.54	2.63	0.94	416.66	3.60	0.92	666.66	3.34	0.92

### Effects of ZnO, MgO, and SiO_2_ NPs and polluted irrigated water containing Cu^2+^ and Fe^3+^ on the soil and plants

#### Effects of ZnO, MgO, and SiO_2_ NPs and polluted irrigated water containing Cu^2+^ on the available concentrations of Cu^2+^ in the soil and plants

The data listed in Table 4 show the effects of adding Cu^2+^at concentrations of 5 and 10 mg kg^−1^ together with different treatments of NPs. It can be clearly seen that the NP application without the addition of any pollutant reduced the available Cu^2+^ in the studied soil samples. The order of the effects of the NP application was as follows: Zn2 > Zn1 > Mg2 > Si2 > Mg 1 > Si1 > C (control). The highest values of the available Cu^2+^ were observed in the control treatment, whereas the lowest values were obtained when Zn2 was added^57^.

**Table 4:** Effect of different NPs and polluted water containing Cu^2+^ and Fe^3+^ on the availability of Cu^2+^ and Fe^3+^ in soil and plant.

Nano exp.	Cu^2+^ additives							
	C	5 mg kg^−1^	10 mg kg^−1^	Mean	C	5 mg kg^−1^	10 mg kg^−1^	Mean
	Cu-DTPA				Cu-plant			
C	4.60F	5.31E	9.37A	6.42A	6.89J	14.30E	17.69B	12.96B
Zn1	1.42I	2.45H	3.40G	2.42E	8.59I	13.34G	8.16I	10.03E
Zn2	0.42J	1.66I	1.58I	1.22F	11.11H	7.39J	14.96D	11.16D
Si1	3.55G	4.11F	8.37B	5.34B	35.71A	13.94EF	11.44H	20.36A
Si2	2.60H	3.37G	7.84C	4.60C	10.96H	13.54FG	2.49M	9.00F
Mg1	3.36G	4.53F	7.98BC	5.29B	7.01J	13.48FG	15.29D	11.93C
Mg2	2.57H	3.42G	6.47D	4.16D	5.31K	3.31L	16.21C	8.28G
Mean	2.64C	3.55B	6.43A		12.23A	11.33B	12.32A	
LSD at 5%	P = 0.197	N = 0.301	P * N = 0.522		P = 0.220	N = 0.336	P * N = 0.582	

Nano exp.	Fe^3+^ additives							
	C	5 mg kg^−1^	10 mg kg^−1^	Mean	C	5 mg kg^−1^	10 mg kg^−1^	Mean
	Fe-DTPA				Fe-plant			
C	12.89G	20.43B	29.56A	20.96A	2087.0L	4404.8B	6277.8A	4256.5A
Zn1	8.61K	10.20IJ	9.177JK	9.33E	1664.5Q	1534.2S	4199.8D	2466.2C
Zn2	5.88L	8.45K	6.75L	7.03F	1629.8R	1779.1P	3025.9H	2144.9G
Si1	11.93GH	19.18BC	19.18BC	16.93B	19698L	3305.9F	4387.3C	3221.0B
Si2	12.88G	14.62F	14.62F	15.23C	2584.8J	1315.2T	2934.8I	2278.3F
Mg1	11.61GH	19.86B	19.86B	15.88C	1899.8M	1863.9O	3347.7E	2370.5E
Mg2	10.64HI	17.29DE	17.29DE	14.16D	24955K	1534.8S	3164.8G	2398.4D
Mean	10.63C	16.71A	16.71A		2047.3C	2248.3B	3905.4A	
LSD at 5%	P = 0.520	N = 0.794	P * N = 1.376		P = 0.8067	N = 1.232	P * N = 2.134	

There was a significant difference between all treatments except for Si2/Mg2 and Si1/Mg1. When Cu^2+^ was added to the soil as an external pollutant to increase the original exit values by 5 mg kg^−1^, the effects of the NP application on the availability of Cu^2+^ extracted using DTPA took the following order: Zn2 > Zn1 > Si2 > Mg2 > Si1 > Mg1 > C (control). The highest value of the effects of NP addition on artificially polluted soils with Cu^2+^ (5 mg kg^−1^) was found when Zn2 was added to the soil, whereas the lowest effect was found when Mg1 was added^58^. There was a significant difference between all treatments except for Si2/Mg2 and Si1/Mg1, similar to what was observed in the control experiments. Increasing the original values of Cu^2+^ by 10 mg kg^−1^ changed the order of effects of the NPs to the following: Zn2 > Zn1 > Mg2 > Si2 > Mg1 > Si1 > C (control).

There was a significant difference between all treatments except for Si2 and Mg1. It can beclearly seen that the highest reduction effect on the available Cu^2+^ in the three trials studied (thecontrol, 5 mg kg^−1^, and 10 mg kg^−1^) was related to the addition of Zn2. The mean values for the three NPs presented significant differences except for Si1 and Mg1. The data presented in Table 4 explore the effects of NPs on Cu^2+^ uptake through plants that were osculated among the higher and lower values compared with the control experiments. The order for increasing Cu^2+^ uptake through plants without the addition of Cu^2+^ was as follows:Si1 > Zn2 > Si2 > Zn1 > Mg1 > C (control) > Mg2.

There was a significant difference between all treatments except for Si2/Zn2 and C(control)/Mg1. When Cu^2+^ was added to the soil at a constant concentration of 5 mg kg^−1^, the order of effects of the NP application was as follows: C (control) > Si1 > Si2 > Mg1 > Zn1 > Zn2 > Mg2. This indicates that the addition of NPs to the soil system decreased Cu^2+^ uptake through plants.

There was a significant difference between all treatments except for Zn1, Si2, and Mg1. Increasing the addition of Cu^2+^ pollutants to the soil to 10 mg kg^−1^ changed the sequence of effects of NP addition to the following order: C (control) > Mg1 > Mg2 > Zn2 > Si1 > Zn1 > Si2. There was a significant difference between all treatments except for Zn1 and Mg2. The application of NPs minimized the Cu^2+^ levels in marjoram because of the antagonistic impacts of metals. The trend of these results agrees with those reported by Saifullah et al.^59^, Hussain et al.^60^, and Wang et al*.*^61^.

#### Effects of ZnO, MgO, and SiO_2_ NPs and polluted irrigated water containing Fe^3+^ on the available concentrations of Fe^3+^ in soil and plant

The effects of adding Fe at concentrations of 5 and 10 mg kg^−1^ with different NP treatments are listed in Table 4. It can be clearly seen that the NP application without the addition of any pollutants reduced the available Fe^3+^ in the soil samples. The order of the effects of the NP application on the reduction of available Fe was found as follows: Zn2 > Zn1 > Mg2 > Mg1 > Si1 > Si2 > C (control)^57^. The highest values of the available Fe^3+^ were observed in the control, whereas the lowest values were obtained when Zn2 was added.

There was a significant difference between all treatments except for Si1 and Mg1. When Fe^3+^ was added to the soil as an external pollutant source to increase the original exit values by 5 mg kg^−1^, the effects of the NPs addition on the availability of Fe^3+^ extracted using DTPA took the following order: Zn2 > Zn1 > Si2 > Mg2 > Si1 > Mg1 > C (control).This is considered similar to what was observed with the same treatment of Cu^2+^. The highest values were found when Zn2 was added to the soil, whereas the lowest values were found when Mg1 was added. There was a significant difference between all treatments except for Mg1 and the control. Increasing the concentration values of Fe^3+^ to 10 mg kg^−1^changed the order of the effects of NP addition to the following: Zn2 > Zn1 > Si2 > Mg2 > Si1 > Mg1 > C (control).

There was a significant difference between all treatments. Clearly, the highest effect in the three trials studied in this work (control, 5 mg kg^−1^, and 10 mg kg^−1^) was related to the addition of Zn2^58,62^. The data presented in Table 4 also explore the effects of NPs on Fe^3+^ uptake through plants that were osculated among the higher and lower values compared with the control experiments. The order of the effects of NP addition on increasing Fe^3+^ uptake through plants was as follows: Si2 > Mg2 > C (control) > Si1 > Mg2 > Zn1 > Zn2.

There was a significant difference between all treatments except for the control and Si1. When Fe^3+^ was added to the soil at a constant concentration of 5 mg kg^−1^, the order of the effects of NP application was as follows: C (control) > Si1 > Mg1 > Zn2 > Mg2 > Zn1 > Si2.Thisindicates that the addition of NPs to the soil system had positive effects on the Cu^2+^ uptake through plants.

There was a significant difference between all treatments except for Mg2 and Zn1. Increasing the addition of Fe^3+^pollutants to the soil to 10 mg kg^−1^ changed the sequence of the effects of NP addition on increasing Fe^3+^ concentrations inside the plant tissue to the following order: C (control) > Si1 > Zn1 > Mg1 > Mg2 > Zn2 > Si2^58,62^.

There was a significant difference between all treatments. At the root surface, NPs can compete with Fe^3+^ as these metals are being transported by common transporters in the roots^63^. Higher Zn concentrations in the roots may inhibit Fe^3+^ uptake by marjoram plants. The trend of these results agrees with those reported by Ali et al.^60^_,_ who suggested that the improved Zn nutritional status with foliar-applied ZnO NPs may suppress the expression of Zn^2+^ transporters in roots, decreasing the Cd^2+^ accumulation by roots. This variation in metal accumulation by plants under NP treatments might be due to the variation in the sizes and shapes of NPs as well as the crop species and NP application modes.

### Effect of Cu^2+^ and Fe^3+^ additives and ZnO, MgO, and SiO_2_ NPs treatment on the availability of N, P, and K in soils

#### Effect of Cu^2+^ additives and ZnO, MgO, and SiO_2_ NPs treatment on the availability of N, P, and K in soils

The effects of Cu^2+^ additives with two concentrations of 5 mg kg^−1^ and 10 mg kg^−1^ simultaneously with different treatment of NPs are listed in Table 5. It was clearly seen that the effects of NPs application on the availability of substantial concentrations of N without any pollutants additives resulted in an osculating in the reduction or increment of the available N in the soil system. The order of sequence for the effects of NPs application on reducing available N was found to take the following sequence Mg2 > Mg1 > C (control) > Si2 > Zn2 > Zn1 > Si1. he highest values that were affected the reduction of the available N were observed with Mg2, whereas the lowest values were obtained when Si1 was added. There was a significant difference between all treatments except for the treatments of (control and Mg1)^58,62^. When Cu^2+^ was added to the soil as an external pollutant to increase the original exit values by 5 mg kg^−1^, the effects of NPs application on the availability of N were found to take the following order Mg2 > Zn2 > Si2 > Si1 > Mg2 > Si2 > C (control) > Zn1. The highest value of the effects of adding NPs on the artificially polluted soils with Cu^2+^ (5 mg kg^−1^) to reduce the availability of N was found when Mg2 was added to the soil, whereas the lowest effect was found when Zn1 was added. There was a significant difference between all treatments except for the treatments of (control and Si2) and (Z2 and Si1). Increasing the values of polluted Cu^2+^ to 10 mg kg^−1^ resulted in changing the order of effects of NPs on the availability of N to the following order Si2 > Mg1 = Mg2 = Si1 = Zn2 > C (control) > Zn1. There was a significant difference between all treatments except for the following treatments Mg1, Mg2, Si1, and Zn2. It was clearly seen that the highest effects in the three trials that were studied in the present work including control, 5 mg kg^−1^, and 10 mg kg^−1^ were osculated among Mg2 and Si2. The effects of Cu^2+^ additives as two concentrations of 5 mg kg^−1^ and 10 mg kg^−1^ simultaneously with different treatment of NPs on available phosphorous (P) are listed in Table 5. It was clearly seen that the effect of NPs application on the availability of substantial concentrations of P without any pollutants additives resulted in an osculating of the reduction or increment of the available P in the soil system. The order of sequence for the effects of NPs application on reducing available P was found to take the following sequence Zn2 > C (control) > Mg2 > Mg1 > Zn1 > Si1 > Si2. The highest values that affected the reduction of the available P were observed with Zn2, whereas the lowest values were obtained when Si2 was added. There was a significant difference between all treatments except for the treatments of (control and Zn2). When Cu^2+^ was added to the soil as an external pollutant to increase the original exit values by 5 mg kg^−1^, the effects of NPs application on reducing the availability of P was found to take the following order Mg2 > Si2 > Mg1 > Zn1 > C (control) > Si1 > Zn2^45,62^. The highest value of the effects of adding NPs on the artificially polluted soils with Cu^2+^ (5 mg kg^−1^) to reduce the availability of P was found when Mg2 was added to the soil, whereas the lowest effect was found when Zn2 was added. There was a significant difference between all treatments except for the treatments of (Si2 and Mg2). Increasing the values of an external pollution of Cu^2+^ to 10 mg kg^−1^ resulted in changing the order of effects of NPs on the availability of P to the following order C (control) > Zn1 > Mg2 > Si1 = Si2 > Mg1 > Zn2. There was a significant difference between all treatments except for the following treatments (Si1 and Si2). It was clearly seen that the highest effects in the three trials that were studied in the present work including control, 5 mg kg^−1^, and 10 mg kg^−1^ were osculated among Zn2, Mg2, and control. The effects of Cu^2+^ additives with two concentrations of 5 mg kg^−1^ and 10 mg kg^−1^ simultaneously with different treatment of NPs on the availability of K are also listed in Table 5. It was clearly seen that the effect of NPs application on the availability of substantial concentrations of K without any pollutants additives resulted in an osculation in the reduction or increment of the available concentrations of available K in the soil system. The order of sequence for the effects of NPs application on reducing the available concentrations of K was found to take the following sequence Mg2 > Si2 > Mg1 > C (control) > Si1 > Zn1 > Zn2. The highest values affected the reduction of the available K were observed with Mg2, whereas the lowest value was obtained when Zn2 was added. There was a significant difference between all treatments except for the treatments of (Si2 and Mg1). When Cu^2+^ was added to the soil as an external pollutant to increase the original exit values by 5 mg kg^−1^, the effects of NPs application on the availability of K were found to take the following order Si1 > Mg1 > Zn2 > C (control) > Mg2 > Zn1 > Si2. The highest value of the effect of adding NPs on the artificially polluted soils with Cu^2+^ (5 mg kg^−1^) to reduce the availability of K was found when Si1 was added to the soil, whereas the lowest effect was found when Si2 was added. Generally, there was a significant difference between all treatments. Increasing the values of an external polluted Cu^2+^ to 10 mg kg^−1^ resulted in changing the order of effects of NPs on the availability of K to the following order Si2 > Si1 > Mg2 > Zn2 > Mg1 = Zn1 > C (control). There was a significant difference between all treatments. It was clearly seen that the highest effects in the three trials that were studied in the present work including control, 5 mg kg^−1^, and 10 mg kg^−1^ on K availability were osculated among Mg2, Si1, and Si2. On the other hand, the lowest effects were attributed to the Zn2 in the control experiment, whereas in the experiments that have been carried out with the addition of Cu^2+^ (5 mg kg^−1^ and 10 mg kg^−1^), the effects of Si2 and control were more dominant. The highest values of available K were found with the control experiment followed by the experiments that were carried out with the addition of an external Cu^2+^ with concentrations of 5 mg kg^−1^ and 10 mg kg^−1^, respectively.

**Table 5:** Effect of polluted water containing Cu^2+^ and Fe^3+^ and different NPs on the availability and accumulation of N, P, and K in the treated soils.

Nano exp.	Cu^2+^											
	C	5 mg kg^−1^	10 mg kg^−1^	Mean	C	5 mg kg^−1^	10 mg kg^−1^	Mean	C	5 mg kg^−1^	10 mg kg^−1^	Mean
	N				P				K			
C	50.73G	121.80C	84.60E	85.71D	25.11G	29.00D	15.52J	23.21D	140.80F	133.74F	178.90D	151.15B
Zn1	135.33B	135.33B	135.33B	135.33A	28.85D	22.23H	17.06I	22.72D	188.27C	209.32B	119.60G	172.39A
Zn2	118.40C	84.60E	67.67F	90.22C	24.78G	32.56AB	32.29AB	29.88A	203.20B	107.91IJ	116.30GH	142.47C
Si1	203.00A	88.00E	67.67F	119.56B	31.61BC	30.81C	26.74E	29.72A	149.80E	87.82K	58.90L	98.84 F
Si2	88.00E	118.40C	50.73G	85.71D	33.09A	14.10K	26.74E	24.64B	114.03GHI	221.75A	40.97M	125.58D
Mg1	50.73G	50.73G	67.67F	56.38E	26.47EF	16.26IJ	28.97D	23.90C	115.17GHI	102.75J	119.60G	112.50E
Mg2	10.53D	101.53D	67.67F	90.24C	25.64FG	13.71K	26.50EF	21.95E	109.07HIJ	155.74E	109.63HIJ	124.81D
Mean	106.82A	100.06B	77.33C		27.93B	22.67C	24.83B		145.76A	145.58A	106.27B	
LSD at 5%	P = 2.5823	N = 3.9446	P * N = 6.8322		P = 0.3989	N = 0.6093	P * N = 1.0554		P = 3.1479	N = 4.8085	P * N = 8.3287	

Nano exp.	Fe^3+^											
	C	5 mg kg^−1^	10 mg kg^−1^	Mean	C	5 mg kg^−1^	10 mg kg^−1^	Mean	C	5 mg kg^−1^	10 mg kg^−1^	Mean
	N				P				K			
C	50.73G	101.50D	84.60E	78.94E	25.11F	29.09C	18.08G	24.09A	140.80IJ	145.60HI	150.37GH	145.59D
Zn1	135.33B	118.40C	67.67F	107.13C	28.85C	26.94D	16.38H	24.06A	188.27D	179.09E	197.83BC	188.40A
Zn2	118.40C	67.67F	84.58E	90.22D	24.78F	15.49H	16.32H	18.86C	203.20AB	135.27J	168.57F	169.01B
Si1	203.00A	101.53D	50.73G	118.42A	31.61B	13.71JK	8.87L	18.06D	149.80H	201.67B	207.60A	186.36A
Si2	88.00E	84.60E	67.67F	80.09E	33.09A	15.28HI	16.23H	21.53B	114.03KL	140.23IJ	199.94B	151.40C
Mg1	50.73G	101.53D	84.60E	78.96E	26.47DE	14.19IJ	12.61K	17.76D	115.17K	194.03C	198.22BC	169.14B
Mg2	101.53D	101.50D	135.33B	112.79B	25.64EF	13.62JK	16.08H	18.45CD	109.07L	155.93G	173.70EF	146.23D
Mean	106.82A	96.68B	82.17C		27.93A	18.33B	14.94C		145.76C	164.55B	185.18A	
LSD at 5%	P = 2.3657	N = 3.6137	P * N = 6.2591		P = 0.4541	N = 0.6937	P * N = 1.2014		P = 2.1487	N = 3.2822	P * N = 5.6849	

#### Effect of Fe^3+^ additives and ZnO, MgO, and SiO_2_ NPs treatment on the availability of N, P, and K in the treated soils

The effects of Fe additives with two concentrations of 5 mg kg^−1^ and 10 mg kg^−1^ simultaneously with different treatment of NPs are listed in Table 6. It was clearly seen that the effect of NPs application on the availability of substantial concentrations of N without any an external Fe additives resulted in an osculating in the reduction or increment of the available N in the soil system. The order of sequence for the effects of NPs application on reducing the available N was found to take the following sequence C (control) = Mg1 > Si1 > Mg2 > Zn2 > Zn1 > Si1. The highest values that affected the reduction of the available N were observed with both control and Mg1, whereas the lowest values were obtained when Si1 was added. There was a significant difference between all treatments except for the treatments of (control and Mg1). When Fe was added to the soil as an external pollutant to increase the original exit values by 5 mg kg^−1^, the effects of NPs application on the reduction of available N were found to take the following order Zn2 > Si2 > Mg2 = C (control) > Si1 = Mg1 > Zn1. The highest value of the effects of adding NPs on the artificially polluted soils with Fe^3+^ (5 mg kg^−1^) to reduce the availability of N was found when Zn2 was added to the soil, whereas the lowest effect was found when Zn1 was added. There was a significant difference between all treatments except for the treatments of (control, Si1, Mg1, and Mg2)^58,62^. Increasing the values of an external addition of Fe^3+^ to 10 mg kg^−1^ resulted in changing the order of effects of NPs on reducing the availability of N to the following order Si1 > Zn1 = Si2 = Zn2 > C (control) > Mg1 > Mg2. There was a significant difference between all treatments except for the following treatments (control and Zn2), (Zn2 and Mg1), and (Zn1 and Si12). It was clearly seen that the highest effect in the three trials that were studied in the present work including control, 5 mg kg^−1^, and 10 mg kg^−1^ was osculated among Mg1, control, Zn2, and Si1. On the other hand, the lowest effects were attributed to the Si1 in the control experiment, whereas in the experiments that have been carried out with the addition of Fe^3+^ with concentrations of 5 mg kg^−1^ 10 mg kg^−1^, the effects of Zn1 and Mg2 were more dominant. The highest values of available N were found with the control experiment followed by experiments that were carried out with the addition of Fe^3+^ at concentrations of 5 mg kg^−1^ and 10 mg kg^−1^. The effects of Fe^3+^ additives with two concentrations of 5 mg kg^−1^ and 10 mg kg^−1^ with different treatment of NPs on the reduction of available P are listed in Table 6. It was clearly seen that the effect of NPs application on the availability of substantial concentrations of P in soil without any pollutants additives resulted in an osculating in the reduction or increment values of the available P in the soil system. The order of sequence for the effects of NPs application on reducing the available P was found to take the following sequence Zn2 > C (control) > Mg2 > Mg1 > Zn1 > Si1 > Si2. The highest values affected the reduction of the available P were observed with Zn2, whereas the lowest values were obtained when Si2 was added. There was a significant difference between all treatments^62^. When Fe^3+^ was added to the soil as an external pollutant to increase the original exit values by 5 mg kg^−1^, the effects of NPs application on the availability of P were found to take the following order Mg2 > Si2 > Mg1 > Si2 > Zn2 > Zn1 > C (control). The highest value of the effects of adding NPs on the artificially polluted soils with Fe^3+^ (5 mg kg^−1^) to reduce the availability of P was found when Mg2 was added to the soil, whereas the lowest effect was found when Zn1 was added. There was a significant difference between all treatments except for the treatments of (Si1 and Mg2). Increasing the values of an external addition of Fe^3+^ to 10 mg kg^−1^ resulted in changing the order of effects of NPs on the availability of P to the following order Si1 > Mg1 > Mg2 > Si2 > Zn2 > Zn1 > C (control). There was a significant difference between all treatments except for the following treatments (Zn1, Zn2, Si2, and Mg2).

**Table 6:** Effect of polluted water containing Cu^2+^ and Fe^3+^ and different NPs on the availability and accumulation of N, P, and K in plant.

Nano exp.	Cu ^2+^											
	C	5 mg kg^−1^	10 mg kg^−1^	Mean	C	5 mg kg^−1^	10 mg kg^−1^	Mean	C	5 mg kg^−1^	10 mg kg^−1^	Mean
	N				P				K			
C	2.783E	3.750C	3.750C	3.428B	0.236DE	0.213GH	0.155K	0.201D	1.003EF	0.903HI	1.100D	1.002C
Zn1	1.453G	2.900E	3.146D	2.500G	0.227EF	0.250B	0.178J	0.218B	0.320L	1.377 C	1.077 D	0.924D
Zn2	3.266D	2.783E	3.266D	3.106C	0.215GH	0.196I	0.065P	0.159F	1.673 B	1.367 C	0.983EFG	1.341A
Si1	2.056F	2.173F	3.866BC	2.699F	0.271A	0.206H	0.220FG	0.238A	0.817J	1.043 DE	0.907 HI	0.922D
Si2	2.880E	3.866BC	4.233A	3.660A	0.246BC	0.240CD	0.134MN	0.206C	0.850IJ	1.8500 A	0.970FG	1.223B
Mg1	2.173F	3.146D	3.266D	2.862E	0.245BC	0.140LM	0.127N	0.171E	0.990 EFG	0.827J	0.950FGH	0.922D
Mg2	3.993B	2.900E	2.173F	3.022D	0.142LM	0.143L	0.113O	0.133G	0.937 GH	1.070D	0.743K	0.916D
Mean	2.658C	3.074B	3.386A		0.226A	0.198B	0.142C		0.941B	1.205 A	0.961B	
LSD at 5%	P = 0.0496	N = 0.0758	P * N = 0.1313		P = 0.0033	N = .00508	P * N = 0.0088		P = 0.0233	N = 0.0356	P * N = 0.0616	

Nano exp.	Fe^3+^											
	C	5 mg kg^−1^	10 mg kg^−1^	Mean	C	5 mg kg^−1^	10 mg kg^−1^	Mean	C	5 mg kg^−1^	10 mg kg^−1^	Mean
	N				P				K			
C	2.783EF	3.506B	3.146D	3.145A	0.236C	0.147J	0.174H	0.186c	1.003E	0.630HIJ	0.643HI	0.758C
Zn1	1.453I	2.900E	2.783EF	2.379D	0.227D	0.142J	0.265A	0.212A	0.320M	2.350A	0.560IJ	1.076A
Zn2	3.266CD	2.660FG	2.056H	2.661C	0.215E	0.162I	0.216E	0.197B	1.673B	0.830G	0.666H	1.056A
Si1	2.056H	3.866A	0.606J	2.177E	0.271A	0.188G	0.077K	0.178D	0.816G	1.260D	0.450KL	0.842B
Si2	2.880E	3.266CD	2.513G	2.887B	0.246B	0.203F	0.190G	0.213A	0.850FG	0.536JK	0.846FG	0.744C
Mg1	2.173H	3.386BC	2.900E	2.820B	0.245B	0.163I	0.232CD	0.213A	0.990E	0.606HIJ	0.363LM	0.653D
Mg2	3.993A	2.660FG	0.363K	2.338D	0.142J	0.170HI	0.077K	0.129E	0.936EF	1.526C	0.203N	0.888B
Mean	2.658B	3.178A	2.053C		0.226A	0.168C	0.176B		0.941B	1.105A	0.533C	
LSD at 5%	P = 0.0632	N = 0.0965	P * N = 0.1671		P = 0.0032	N = 0.0049	P * N = 0.0086		P = 0.0358	N = 0.0547	P*N = 0.0947	

It was clearly seen that the highest effect in the three trials that were studied in the present work including control, 5 mg kg^−1^, and 10 mg kg^−1^ was osculated among Zn2, Mg2, and Si1. The effects of Fe^3+^ additives with two concentrations of 5 mg kg^−1^ and 10 mg kg^−1^ simultaneously with different treatment of NPs on the reduction of available K are listed in Table 6. It was clearly seen that the effect of NPs application on the availability of substantial concentrations of K in soil without any pollutants additives resulted in an osculating in the reduction or increment of the available concentrations of K in the soil system. The order of sequence for the effects of NPs application on reducing the available concentrations of K was found to take the following sequence Mg2 > Si2 > Mg1 > C (control) > Zn1 > Si1 > Zn2. The highest values affected the reduction of available K were observed with Mg2, whereas the lowest values were obtained when Zn2 was added^58,62^. There was a significant difference between all treatments. When Fe^3+^ was added to the soil as an external pollutant to increase the original exit values by 5 mg kg^−1^, the effects of NPs application on the availability of K was found to take the following order Zn2 > Si2 > C (control) > Mg2 > Zn1 > Mg1 > Si1. The highest value of the effects of adding NPs on the artificially polluted soils with Fe^3+^ (5 mg kg^−1^) to reduce the availability of K was found when Zn2 was added to the soil, whereas the lowest effect was found when Si1 was added. There was a significant difference between all treatments except for (Mg1 and Mg2). Increasing the values of an external addition of Fe^3+^ to 10 mg kg^−1^ resulted in changing the order of effects of NPs on the availability of K to the following order C (control) > Zn2 > Mg2 > Zn1 > Mg1 > Si2 > Si1. There was a significant difference between all treatments except for (Z1 and Mg1)^62^. It was clearly seen that the highest effects in the three trials that were studied in the present work including control, 5 mg kg^−1^, and 10 mg kg^−1^ on K availability were osculated among Mg2, Zn2, and control.

### Effect of Cu^2+^ and Fe^3+^ additives and ZnO, MgO, and SiO_2_ NPs treatment on the accumulation of N, P, and K in the plant

#### Effect of Cu^2+^ additives and ZnO, MgO, and SiO_2_ NPs treatment on the accumulation of N, P, and K in the plant

The effects of Cu^2+^ additives with two concentrations of 5 mg kg^−1^ and 10 mg kg^−1^ simultaneously with different treatment of NPs on increasing the total amounts of N in the plant are listed in Table 6. It was clearly seen that the effect of NPs application on increasing the total concentrations of N without any Cu^2+^ additives resulted in an osculation in the reduction or increment of N in the plant^62^. The order of sequence for the effects of NPs application on the increasing the total N was found to take the following sequence Mg2 > Zn2 > Si2 > Si1 > C (control) > Zn2 > Zn1 > Si1. The highest values affected the increases of the available N in the plant were observed with Mg2, whereas the lowest values were obtained when Si1 was added. There was a significant difference between all treatments except for the treatments of (control and Si2) and (Si1 and Mg1). When Cu^2+^ was added to the soil as an external pollutant to increase the original exit values by 5 mg kg^−1^, the effects of NPs application on the increment of total N were found to take the following order Si2 > C (control) > Mg1 > Zn1 = Mg2 > Zn2 > Si1. The highest value of the effects of adding NPs on the artificially polluted soils with Cu^2+^ (5 mg kg^−1^) to increase the total concentrations of N in the plant was found when Si2 was added to the soil, whereas the lowest effect was found when Si1 was added^62^. There was a significant difference between all treatments except for the treatments of (control and Si2) and (Z1 and Mg2). Increasing the values of an external addition of Cu2 + to 10 mg kg^−1^ resulted in changing the order of effects of NPs on increase the total concentrations of N in the plant to the following order Si2 > Si1 > C (control) > Zn2 = Mg1 > Zn1 > Mg2. There was a significant difference between all treatments except for the following treatments (control and Si1) and (Zn1, Zn2, and Mg1). It was clearly seen that the highest effects in the three trials that were studied in the present work including control, 5 mg kg^−1^, and 10 mg kg^−1^ were osculated among Mg2, Si2, and Si2, respectively. The effects of Cu^2+^ additives with two concentrations of 5 mg kg^−1^ and 10 mg kg^−1^ simultaneously with different treatment of NPs on increasing the total amounts of P in the plant are listed in Table 6. It was clearly seen that the effect of NPs application on increasing the total concentrations of P without any pollutants additives resulted in an osculating in the reduction or increment of P in the plant. The order of sequence for the effects of NPs application on the increasing the total P was found to take the following sequence Si1 > Si2 > Mg1 > C (control) > Zn1 > Zn2 > Mg2. The highest values affected the increases of the available P were observed with Si1, whereas the lowest values were obtained when Mg2 was added. There was a significant difference between all treatment except for the treatments of (Si2 and Mg1). When Cu^2+^ was added to the soil as a pollutant to increase the original exit values by 5 mg kg^−1^, the effects of NPs application on the increment of total P were found to take the following order Zn1 > Si2 > C (control) > Si1 > Zn2 > Mg2 > Mg1^62^. The highest value of the effects of adding NPs on the artificially polluted soils with Cu^2+^ (5 mg kg^−1^) to increase the total concentrations of P in the plant was found when Zn1 was added to the soil, whereas the lowest effect was found when Mg1 was added. There was a significant difference between all treatments. Increasing the values of an external addition of Cu^2+^ to 10 mg kg^−1^ resulted in changing the order of effects of NPs on increase the total concentrations of P in the plant to the following order Si1 > Zn1 > C (control) > Si2 > Mg1 > Mg2 > Zn2. There was a significant difference between all treatments. It was clearly seen that the highest effects in the three trials that were studied in the present work including the control, 5 mg kg^−1^, and 10 mg kg^−1^ were osculated among Si1, Zn1, and Si1, respectively. The effects of Cu^2+^ additives with two concentrations of 5 mg kg^−1^ and 10 mg kg^−1^ simultaneous with different treatments of NPs on increasing the total amounts of K in the plant are listed in Table 6. It was clearly seen that the effect of NPs applications on increasing the total concentrations of K without any Cu^2+^ additives were resulted in an osculating in the reduction or increment of total K in the plant. The order of sequence for the effects of NPs application on increasing the total K was found to take the following sequence Zn2 > C (control) > Mg1 > Mg2 > Si2 > Si1 > Zn1. The highest values affected the increases of the total N were observed with Zn2, whereas the lowest values were obtained when Zn1 was added. There was a significant difference between all treatments. When Cu^2+^ was added to the soil as an external pollutant to increase the original exit values by 5 mg kg^−1^, the effects of NPs application on the increment of total K were found to take the following order Si2 > Zn1 > Zn2 > Mg2 > Si1 > C (control) > Mg1. The highest values of the effects of adding NPs on the artificially polluted soils with Cu^2+^ (5 mg kg^−1^) to increase the total concentrations of K in the plant were found when Si2 was added to the soil, whereas the lowest effect was found when Mg1 was added. There was a significant difference between all treatments except for the treatments of (Z1 and Zn2). Increasing the values of Cu^2+^ to 10 mg kg^−1^ resulted in changing the order of effects of NPs on increase the total concentrations of K in the plant to the following order C (control) > Zn1 > Zn2 > Si2 > Mg1 > Si1 > Mg2^58,62^. There was a significant difference between all treatments except for the following treatments (control and Zn1). It was clearly seen that the highest effects in the three trials that were studied in the present work including control, 5 mg kg^−1^, and 10 mg kg^−1^ were osculated among Zn2, Si2, and control, respectively.

#### Effect of Fe^3+^ additives and ZnO, MgO, and SiO_2_ NPs treatment on the accumulation of N, P, and K in the plant

The effects of Fe^3+^ additives with two concentrations of 5 mg kg^−1^ and 10 mg kg^−1^ simultaneously with different treatment of NPs on increasing the total amounts of N in the plant are listed in Table 6. It was clearly seen that the effect of NPs application on increasing the total concentrations of N without any pollutants additives resulted in an osculating in the reduction or increment of N in the plant. The order of sequence for the effects of NPs application on the increasing the total N was found to take the following sequence Mg2 > Zn2 > Si2 > C (control) > Mg1 > Si1 > Zn1. The highest values affected the increases of the total N were observed with Mg2, whereas the lowest values were obtained when Zn1 was added. There was a significant difference between all treatment except for the treatments of (Si1 and Mg1). When Fe^3+^ was added to the soil as an external addition to increase the original exit values by 5 mg kg^−1^, the effects of NPs application on the increment of total N were found to take the following order Si1 > C (control) > Mg1 > Si1 > Zn1 > Zn2 = Mg2. The highest value of the effects of adding NPs on the artificially polluted soils with Fe^3+^ (5 mg kg^−1^) to increase the total concentrations of N in the plant was found when Si1 was added to the soil, whereas the lowest effect was found when Mg2 was added. There was a significant difference between all treatments except for the treatments of (Zn2 and Mg2). Increasing the values of polluted Fe^3+^ to 10 mg kg^−1^ resulted in changing the order of effects of NPs on increase the total concentrations of N in the plant to the following order C (control) > Mg1 > Zn1 > Si2 > Zn2 > Si1 > Mg2. There was a significant difference between all treatments. It was clearly seen that the highest effects in the three trials that were studied in the present work including control, 5 mg kg^−1^, and 10 mg kg^−1^ were osculated among Mg2, Si1, and control, respectively. The effects of Fe^3+^ additives with two concentrations of 5 mg kg^−1^ and 10 mg kg^−1^ simultaneously with different treatment of NPs on increasing the total amounts of P in the plant are listed in Table 6. It was clearly seen that the effect of NPs application on increasing the total concentrations of P without any pollutants additives resulted in an osculating in the reduction or increment of P in the plant^58^. The order of sequence for the effects of NPs application on the increasing the total P was found to take the following sequence Si1 > Si2 > Mg1 > C (control) > Zn1 > Zn2 > Mg2 which is similar to what was observed with Cu^2+^ additives at the same concentrations. The highest values affected the increases of the total P were observed with Si1, whereas the lowest values were obtained when Mg2 was added. There was a significant difference between all treatments except for the treatments of (Si2 and Mg1). When Fe^3+^ was added to the soil as pollutant to increase the original exit values by 5 mg kg^−1^, the effects of NPs application on the increment of total P were found to take the following order Si2 > Si1 > Mg2 > Mg1 > Zn2 > C (control) > Si1 = Mg2. The highest values of the effects of adding NPs on the artificially polluted soils with Fe^3+^ (5 mg kg^−1^) to increase the total concentrations of P in the plant were found when Si2 was added to the soil, whereas the lowest effects were found when Mg2 were added. There was a significant difference between all treatments except for (control and Zn1) and (Zn2 and Mg1). Increasing the values of an external addition of Fe^3+^ to 10 mg kg^−1^ resulted in changing the order of effects of NPs on increasing the total concentrations of P in the plant to the following order Zn1 > Mg1 > Zn2 > Si2 > C (control) > Si1 = Mg2^62^. There was a significant difference between all treatments. It was clearly seen that the highest effects in the three trials that were studied in the present work including control, 5 mg kg^−1^, and 10 mg kg^−1^ were osculated among Si1, Si2, and Zn1, respectively. The effects of Fe^3+^ additives with two concentrations of 5 mg kg^−1^ and 10 mg kg^−1^ simultaneously with different treatment of NPs on increasing the total amounts of K in the plant are listed in Table 6. It was clearly seen that the effect of NPs application on increasing the total concentrations of K without any pollutants additives resulted in an osculating in the reduction or increment of K in the plant. The order of sequence for the effects of NPs application on increasing the total K was found to take the following sequence Zn2 > C (control) > Mg1 > Mg2 > Si2 > Si1 > Zn1 which is similar to what was observed during Cu^2+^ additives at the same concentrations. The highest values affected the increases of the total K were observed with Zn2, whereas the lowest values were obtained when Zn1 was added. There was a significant difference between all treatments except for (control and Mg1). When Fe^3+^ was added to the soil as an external addition to increase the original exit values by 5 mg kg^−1^, the effects of NPs application on the increment of total K were found to take the following order Zn1 Mg2 > Si1 > Zn2 > C (control) > Mg1 > Si2. The highest value of the effects of adding NPs on the artificially polluted soils with Fe^3+^ (5 mg kg^−1^) to increase the total concentrations of K in the plant was found when Zn1 was added to the soil, whereas the lowest effect was found when Si2 was added. There was a significant difference between all treatments except for the treatments of (control and Mg1)^58^. Increasing the values of an external addition of Fe^3+^ to 10 mg kg^−1^ resulted in changing the order of effects of NPs on increase the total concentrations of K in the plant to the following order Si2 > Zn2 > C (control) > Zn1 > Si1 > Mg1 > Mg2. There was a significant difference between all treatments. It was clearly seen that the highest effects in the three trials that were studied in the present work including control, 5 mg kg^−1^, and 10 mg kg^−1^ were osculated among Zn2, Zn1, and Si2, respectively. The trend of these results agreed with those reported by Hegab et al.^63,64^ and Ali et al.^61^.

### Effect of different ZnO, MgO, and SiO_2_ NPs and polluted water containing Cu^2+^ and Fe^3+^ solution on dry and wet weights of Marjoram plant.

#### Effect of different ZnO, MgO, and SiO_2_ NPs and polluted water containing Cu^2+^ on dry and wet weights of Marjoram plant

The effects of Cu^2+^ additives with different NPs treatments on dry and wet weights of Marjoram are presented in Table 7. The effects of two concentrations (5 mg kg^−1^ and 10 mg kg^−1^) of Cu^2+^ additives have been investigated. It was clearly seen that the effect of NPs application presented a negative effect on Marjoram dry weight in the experiments that have been carried out without Cu^2+^ additives. The order of sequence for the effects of NPs application on increasing the dry weight was found to take the following order control > Si2 = Mg1 > Si1 > Zn1 > Mg2 > Zn2. The highest values of means for dry weight were observed in the control treatment, whereas the lowest values were obtained when Zn2 was added. There was a significant difference between all treatments except for the treatments of (Si2 and Mg2)^62^. When Cu^2+^ was added to the soil as an external addition to increase the original exit values by 5 mg kg^−1^, the effects of NPs application on Marjoram dry weight took the following order Zn2 > Mg2 > Si2 > C (control) > Zn1 > Si1 > Mg1. The highest value of the effects of adding NPs on the artificially polluted soils with Cu^2+^ (5 mg kg^−1^) was found when Zn2 was added to the soil, whereas the lowest effect was found when Mg1 was added. There was a significant difference between all treatments except for the treatments of (control, Zn1, and Si2) and (Si1 and Mg1). Increasing the values of original exist values of Cu^2+^ by 10 mg kg^−1^ resulted in changing the order of effects of NPs on Marjoram dry weight to the following order Zn1 > Zn2 > Mg2 > Si2 > C (control) > Si1 Mg1. There was a significant difference between all treatments except for the treatments of (control, Si1, Si2 and Mg1) and (Zn2, Mg2). It was clearly seen that the highest effect in the three trials that were studied in the present work including control, 5 mg kg^−1^, and 10 mg kg^−1^ was relevant to the additives of control, Zn2, and Zn1, respectively. It was clearly seen that the effect of NPs application presented a negative effect on Marjoram wet weight in the experiments that have been carried out without Cu^2+^ additives. The order of sequence for the effects of NPs application on increasing the wet weight was found to take the following order control > Si2 = Mg1 > Si1 > Mg2 > Zn1 > Zn2. The highest values of means for wet weight were observed in the control treatment, whereas the lowest values were obtained when Zn2 was added which is similar to data that has been observed in dry weight. There was a significant difference between all treatments except for the treatments of Si2 and Mg1. When Cu^2+^ was added to the soil as an external pollutant to increase the original exiting values by 5 mg kg^−1^, the effects of NPs application on Marjoram wet weight took the following order Zn2 > Si1 = Mg2 > Zn1 = Mg1 > C (control) > Si2. The highest value of the effects of adding NPs on the artificially polluted soils with Cu^2+^ (5 mg kg^−1^) was found when Zn2 was added to the soil, whereas the lowest effect was found when Si2 was added. There was a significant difference between all treatments except for the treatments of control, Zn1, Si1, Mg1, and Mg2. Increasing the values of original exist values of Cu^2+^ addition by 10 mg kg^−1^ resulted in changing the order of effects of NPs on Marjoram wet weight to the following order Zn1 > Zn2 > C (control) > Mg1 > Si2 = Mg2 > Si1. There was a significant difference between all treatments except for the treatments of (control and Zn2) and (Si2, Mg1, and Mg2). It was clearly seen that the highest effects in the three trials that were studied in the present work including control, 5 mg kg^−1^, and 10 mg kg^−1^ were relevant to the additives of control, Zn2, and Zn1, respectively similar to what was observed in dry weight. The fresh and dry weights were affected by the use of Zn, Si, Mg NPs and may be the lower biomass in the control plants might be due to the higher Fe^3+^ and Cu^2+^ levels in these plants. These elements mediated reduction in plant growth might be associated with the disturbance of several mechanisms in plants such as chlorophyll biosynthesis, water deficit, and ultra-structural alteration in plant^65^. These results are in close conformity with Venkatachalam et al.^66^.

**Table 7:** Effect of different NPs and polluted water on dry and wet weights of Marjoram plant.

NPs treatments	Cu^2+^							
	C	5 mg kg^−1^	10 mg kg^−1^	Mean	C	5 mg kg^−1^	10 mg kg^−1^	Mean
	Dry weight				Wet weight			
C	36.96A	15.30DEF	14.96DEF	22.41A	51.66A	25.33DEF	25.66DEF	34.22A
Zn1	17.96CDE	14.30DEF	18.96CD	17.07BC	22.66EF	25.66DEF	36.66B	28.33B
Zn2	11.96F	17.96CDE	16.96CDE	15.63C	20.33F	32.66BC	26.66DEF	26.55B
Si1	20.96BC	13.63EF	14.30DEF	16.41BC	30.66BCD	26.00DEF	20.66F	25.77B
Si2	23.96B	15.96DEF	15.63DEF	18.52B	32.66BC	22.00EF	22.66EF	25.77B
Mg1	23.96B	13.63EF	14.30DEF	17.30BC	32.66BC	25.66DEF	23.33EF	27.22B
Mg2	17.96CDE	17.30CDE	16.96CDE	17.41BC	27.66CD	26.00DEF	22.66EF	25.44B
Mean	21.96A	15.49B	16.01B		31.19A	26.19B	25.47B	
LSD at 5%	P = 1.82	N = 2.78	P * N = 4.83		P = 2.41	N = 3.68	P * N = 6.38	

NPs treatments	Fe^3+^							
	C	5 mg kg^−1^	10 mg kg^−1^	Mean	C	5 mg kg^−1^	10 mg kg^−1^	Mean
	Dry weight				Wet weight			
C	36.96B	19.96EF	19.96EF	25.63B	51.66B	32.66EF	38.66D	41.00B
Zn1	17.96FG	16.96G	16.96G	17.30D	22.66IJ	22.66IJ	26.00HI	23.77E
Zn2	11.96H	19.96EF	19.96EF	17.30D	20.33J	32.66EF	32.66EF	28.55D
Si1	20.96DE	22.96CD	15.30G	19.74C	30.66FG	35.66DE	22.00IJ	29.44D
Si2	23.96C	15.63G	34.96B	24.85B	32.66EF	28.00GH	52.66B	37.77C
Mg1	23.96C	23.96C	65.96A	37.96A	32.66EF	43.66C	94.66A	57.00A
Mg2	17.96G	15.96G	19.96EF	17.96D	27.66GH	21.66J	32.66EF	27.33D
Mean	21.96B	19.34C	27.58A		31.19B	31.00B	42.76A	
LSD at 5%	P = 1.09	N = 1.67	P * N = 2.90		P = 1.62	N = 2.48	P * N = 4.29	

#### Effect of ZnO, MgO, and SiO_2_ NPs and polluted water containing Fe^3+^ on dry and wet weights of Marjoram plant

The effects of Fe^3+^ additives with different NPs treatments on dry and wet weights of Marjoram are presented in Table 7. The effects of two concentrations of 5 mg kg^−1^ and 10 mg kg^−1^ of Feon dry and wet weights of Marjoram plant have been investigated. It was clearly seen that the effect of NPs application presented a negative effect on Marjoram dry weight in the experiments that have been carried out without Fe^3+^ additives. The order of sequence for the effects of NPs application was found to take the following order C (control) > Si2 = Mg1 > Si1 > Zn1 > Mg2 > Zn2. The highest values of means for dry weight were observed in the control treatment, whereas the lowest values were obtained when Zn2 was added. There was a significant difference between all treatments except for the treatments of Si2 and Mg1. When Fe^3+^ was added to the soil as an external addition to increase the original exit values by 5 mg kg^−1^, the effects of NPs application on Marjoram dry weight took the following order Mg1 > Si1 > C (control) = Zn2 > Zn1 > Mg2 > Si2. The highest value of the effects of adding NPs on the artificially polluted soils with Fe^3+^ (5 mg kg^−1^) was found when Mg1 was added to the soil, whereas the lowest effect was found when Si2 was added. There was a significant difference between all treatments except for the treatments of (control and Zn2) and (Zn1, Si2, and Mg2)^54,58^. Increasing the values of original exist values of Fe^3+^ by 10 mg kg^−1^ resulted in changing the order of effects of NPs on Marjoram dry weight to the following order Mg1 > Si2 > C (control) = Zn2 = Mg2 > Zn1 > Si1. There was a significant difference between all treatment except for the treatments of (control, Zn2, and Mg2) and (Zn1 and Si1). It was clearly seen that the highest effects in the three trials that were studied in the present work including control, 5 mg kg^−1^, and 10 mg kg^−1^ were relevant to the additives of control, Mg1, and Mg1, respectively. It was clearly seen that the effect of NPs application presented a negative effect on Marjoram wet weight in the experiments that have been carried out without Fe^3+^ additives. The order of sequence for the effects of NPs application was found to take the following order C (control) > Si2 = Mg1 > Si1 > Mg2 > Zn1 > Zn2. The highest values of means for wet weight were observed in the control treatment, whereas the lowest values were obtained when Zn2 was added. There was a significant difference between all treatments except for the treatments of (Si2 and Mg1). When Fe^3+^ was added to the soil as an external addition to increase the original exit values by 5 mg kg^−1^, the effects of NPs application on Marjoram wet weight took the following order Mg1 > Si1 > control = Zn2 > Si2 > Zn1 > Mg2. The highest values of the effects of adding NPs on the artificially polluted soils with Fe^3+^ (5 mg kg^−1^) were found when Mg1 was added to the soil, whereas the lowest effect was found when Mg2 was added. There was a significant difference between all treatments except for the treatments of control and Zn2. Increasing the values of original exist values of Fe^3+^ by 10 mg kg^−1^ resulted in changing the order of effects of NPs on Marjoram wet weight to the following order Mg1 > Si2 > C (control) > Zn2 > Mg2 > Zn1 > Si1. There was a significant difference between all treatment except for the treatments of (Zn2 and Mg2). It was clearly seen that the highest effect in the three trials that were studied in the present work including control, 5 mg kg^−1^, and 10 mg kg^−1^ was relevant to the additives of control, Mg1, and Si2, respectively. These results are in close conformity with Tripathi et al.^67^ who reported that Supply of SiO_2_ NPs enhanced the photosynthetic pigments in Cr-stressed pea leaves.

## Conclusion

In this study, we conducted a green synthesis of ZnO, MgO, and SiO_2_ NPs to investigate their effects on the quality of irrigation water, the availability of some heavy metals in soil and plants, and the productivity of marjoram. The results obtained from our experiments explored that the addition of NPs resulted in noticeable variations in the removal percentages of both Cu^2+^and Fe^3+^. The maximum values obtained for the adsorption of Cu^2+^ on ZnO, MgO, and SiO_2_ NPs within the pH values of 3–5 were 89.9%, 83.3%, and 68.36%, respectively. In contrast, the maximum adsorption values of Fe^3+^ at pH 3.3 were 82%, 80%, and 65% for the ZnO, MgO, and SiO_2_ NPs, respectively.

Whereas, the maximum adsorption values of Fe^3+^ at pH 3.3 were 82%, 80%, and 65% for ZnO, MgO, and SiO_2_ NPs, respectively. Whereas, the maximum adsorption values of Fe^3+^ at pH 3.3 were 82%, 80%, and 65% for ZnO, MgO, and SiO_2_ NPs, respectively. It was clearly seen that the effect of NPs application on the availability of substantial concentrations of Cu^2+^ without any pollutants additives resulted in the reduction of the available Cu^2+^ in the soil samples, and that same tendency was observed with the substantial concentrations of Fe exist in the soil. Adding NPs to the soil system had positive effects on Cu^2+^ uptake via the plant. The effect of NPs and the addition of Cu^2+^ and Fe^3+^ on the availability of NPK in the soil system were very completed and osculated from one treatment to another. The same tendency was observed with the total concentration of NPK in the plant. It was clearly seen that the effects of NPs application have presented a negative effect on Marjoram dry and wet weight in the experiments that have been carried out without the additions of either Cu^2+^ or Fe^3+^.
